# Sigma-1 Receptor Promotes Mitochondrial Bioenergetics by Orchestrating ER Ca^2+^ Leak during Early ER Stress

**DOI:** 10.3390/metabo11070422

**Published:** 2021-06-26

**Authors:** Zhanat Koshenov, Furkan E. Oflaz, Martin Hirtl, Johannes Pilic, Olaf A. Bachkoenig, Benjamin Gottschalk, Corina T. Madreiter-Sokolowski, Rene Rost, Roland Malli, Wolfgang F. Graier

**Affiliations:** 1 Molecular Biology and Biochemistry, Gottfried Schatz Research Center, Medical University of Graz, Neue Stiftingtalstraße 6/6, 8010 Graz, Austria; zhanat.koshenov@medunigraz.at (Z.K.); furkan.oflaz@medunigraz.at (F.E.O.); martin.hirtl@medunigraz.at (M.H.); johannes.pilic@medunigraz.at (J.P.); olaf.bachkoenig@medunigraz.at (O.A.B.); benjamin.gottschalk@medunigraz.at (B.G.); corina.madreiter@medunigraz.at (C.T.M.-S.); rene.rost@medunigraz.at (R.R.); roland.malli@medunigraz.at (R.M.); 2 BioTechMed Graz, Mozartgasse 12/II, 8010 Graz, Austria

**Keywords:** sigma-1 receptor, mitochondrial bioenergetics, ER stress, UPR, ER Ca^2+^ leak, mitochondrial Ca^2+^, mitochondrial metabolism

## Abstract

The endoplasmic reticulum (ER) is a complex, multifunctional organelle of eukaryotic cells and responsible for the trafficking and processing of nearly 30% of all human proteins. Any disturbance to these processes can cause ER stress, which initiates an adaptive mechanism called unfolded protein response (UPR) to restore ER functions and homeostasis. Mitochondrial ATP production is necessary to meet the high energy demand of the UPR, while the molecular mechanisms of ER to mitochondria crosstalk under such stress conditions remain mainly enigmatic. Thus, better understanding the regulation of mitochondrial bioenergetics during ER stress is essential to combat many pathologies involving ER stress, the UPR, and mitochondria. This article investigates the role of Sigma-1 Receptor (S1R), an ER chaperone, has in enhancing mitochondrial bioenergetics during early ER stress using human neuroblastoma cell lines. Our results show that inducing ER stress with tunicamycin, a known ER stressor, greatly enhances mitochondrial bioenergetics in a time- and S1R-dependent manner. This is achieved by enhanced ER Ca^2+^ leak directed towards mitochondria by S1R during the early phase of ER stress. Our data point to the importance of S1R in promoting mitochondrial bioenergetics and maintaining balanced H_2_O_2_ metabolism during early ER stress.

## 1. Introduction

Among a wide array of functions attributed to the endoplasmic reticulum (ER), its protein folding and Ca^2+^ signaling capabilities are perhaps the most studied ones. These functions require meticulous control and regulation, where disturbance of either can result in ER stress with serious pathological outcomes [1]. ER stress represents an inability of ER to properly fold and process proteins in its lumen, resulting in their accumulation and disruption of regular ER functions. There are three main proteins that sense and act upon ER stress, namely, double-stranded RNA-dependent protein kinase (PRK)-like ER kinase (PERK), activating transcription factor 6 (ATF6), and inositol requiring enzyme 1 (IRE1), which form the core of ER unfolded protein response (UPR) [2]. The primary function of UPR is to restore ER homeostasis following ER stress and involves halt of new protein synthesis, while increasing ER chaperone gene expression. These processes are accompanied by the removal of unfolded proteins (ERAD) from the ER lumen [3]. Importantly, since UPR is energetically demanding, the ER requires more ATP, mainly supplied by mitochondria [4]. Hence, it has been reported that ER-mitochondria tethering is increased during the early stages of ER stress to exchange Ca^2+^ more efficiently and to promote mitochondrial ATP production [5]. Nevertheless, prolonged ER stress and UPR were shown to lead to apoptosis, due to mitochondrial Ca^2+^ overload [6]. 

Sigma-1 receptor (S1R), an ER chaperone mainly residing in mitochondria-associated ER membranes (MAMs), was implicated in both Ca^2+^ signaling and cell survival during ER stress [7]. Under ER stress, S1R was shown to interact with several prominent ER proteins, including inositol 1,4,5-trisphosphate receptor 3 (IP_3_R3) and IRE1. Association of S1R with IP_3_R3 was shown to stabilize the latter at the MAMs and increase ER-mitochondrial Ca^2+^ signaling [7], whereas interaction of S1R with IRE1 was shown to sustain prolonged UPR by IRE1 [8]. Numerous reports demonstrated the involvement of ER stress and S1R deficiency in various neurodegenerative diseases [9,10] and cancer [11], emphasizing the importance of elucidating the exact role of S1R in ER stress. In this work, we scrutinize the involvement of S1R in promoting mitochondrial bioenergetics during the early phases of ER stress by imaging mitochondrial and ER activities in single cells with high-resolution fluorescence microscopy. Using SH-SY5Y neuroblastoma cells, we have been able to demonstrate that S1R is indispensable for increased mitochondrial bioenergetics during early ER stress. We could show that this is achieved by an ER Ca^2+^ leak that is directed towards mitochondria by S1R. 

## 2. Results

### 2.1. XBP1 Splicing Increases during Early ER Stress and Is Not Affected by Sigma-1 Receptor Knock-Down

**Figure 1:**
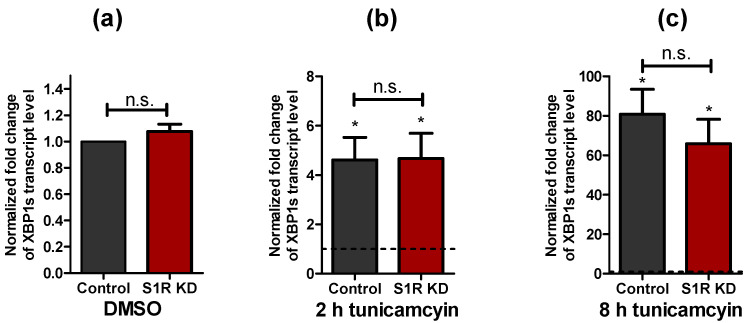
Impact of tunicamycin treatment on XBP1 splicing levels. Bar graphs represent mean ± SEM of spliced XBP1 transcript levels in control and S1R KD SH-SY5Y neuroblastoma cells under control conditions (**a**) or after tunicamycin treatment for 2 h (**b**) and 8 h (**c**). Dashed lines represent the transcript level of spliced XBP1 in the respective DMSO treated cells. Paired t-test, n = 3, * *p* < 0.05 against corresponding DMSO treated cells; unpaired t-test between control and S1R KD, n = 3, n.s.—not significant.

To induce ER stress, SH-SY5Y neuroblastoma cells were treated with 0.6 µM tunicamycin for 2 h and 8 h. The X-box binding protein 1 (XBP1) splicing by IRE1, a well-established marker of ER stress [12,13], was not changed by S1R knock-down (S1R KD), while it was increased in cells treated with tunicamycin for 2 h that dramatically increased upon 8 h treatment (Figure 1a–c). 

Since the majority of the experiments in this work were performed using single cells co-transfected with a genetically encoded sensor and siRNA, we quantified the knock-down efficiency in co-transfected GFP-positive sorted cells, which showed much higher knock-down efficiency compared to unsorted cells (Figure S1a,b).

### 2.2. Sigma-1 Receptor Is Essential for Increased Mitochondrial ATP Level during Early ER Stress

**Figure 2:**
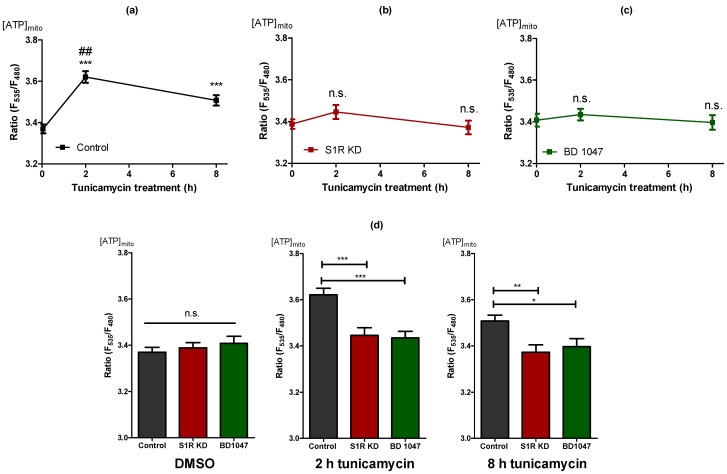
The increase in mitochondrial ATP levels during early ER stress is dependent on S1R. Time course of mitochondrial ATP levels after tunicamycin treatment, presented as mean ± SEM and assessed by the mitochondrial-targeted ATP biosensor AT1.03 in control SH-SY5Y cells (**a**), in cells with S1R KD (**b**) or after treatment with BD 1047 (**c**). (**d**) Bar graphs represent MEAN ± SEM of mitochondrial ATP levels in control (black), S1R KD (red), and BD1047 treated (green) cells before tunicamycin treatment (left), after 2 h (middle), or 8 h (right) tunicamycin treatment. One-way ANOVA with Tukey’s multiple comparison test, * *p* < 0.05, ** *p* < 0.01, *** *p* < 0.001, ## *p* < 0.001 (comparison between control cells after 2 h and 8 h tunicamycin treatment), n.s.—not significant; Control DMSO (174 cells/18 experiments), Control 2 h tunicamycin (97 cells/10 experiments), Control 8 h tunicamycin (120 cells/11 experiments), S1R KD DMSO (141 cells/15 experiments), S1R KD 2 h tunicamycin (67 cells/7 experiments), S1R KD 8 h tunicamycin (74 cells/8 experiments), BD1047 DMSO (93 cells/9 experiments), BD1047 2 h tunicamycin (80 cells/7 experiments), BD1047 8 h tunicamycin (100 cells, 9 experiments).

To assess the impact of S1R on mitochondrial bioenergetics during the early phases of ER stress, we measured mitochondrial ATP levels of SH-SY5Y cells using a genetically encoded mitochondria-targeted ATP probe, mtAT1.03 [14] after 2 h and 8 h of tunicamycin treatment. Mitochondrial ATP was maximally increased after 2 h and slightly after 8 h of tunicamycin treatment of control cells (Figure 2a). Basal mitochondrial ATP levels remained unaffected in cells treated with siRNA against S1R (Figure 2a,b). The mitochondrial ATP increases in response to tunicamycin at both time points were, however, abolished by S1R knock-down (Figure 2b), indicating the involvement of S1R in raising mitochondrial ATP during tunicamycin-induced ER stress. To further validate these results, we have used a selective S1R antagonist, BD1047 [15]. Similar to the knock-down of S1R, BD1047 did not affect basal ATP levels within mitochondria, but greatly attenuated the mitochondrial ATP elevations in response to 2 h and 8 h tunicamycin treatment (Figure 2c), thus supporting the need for S1R to increase mitochondrial ATP during early ER stress. Interestingly, neither knock-down nor BD1047 affected mitochondrial ATP levels without tunicamycin treatment (Figure 2d), implying the importance of S1R for mitochondrial ATP production primarily during ER stress.

### 2.3. Sigma-1 Receptor Is Promoting Mitochondrial Hyperpolarisation and NADH Consumption during Early ER Stress

**Figure 3:**
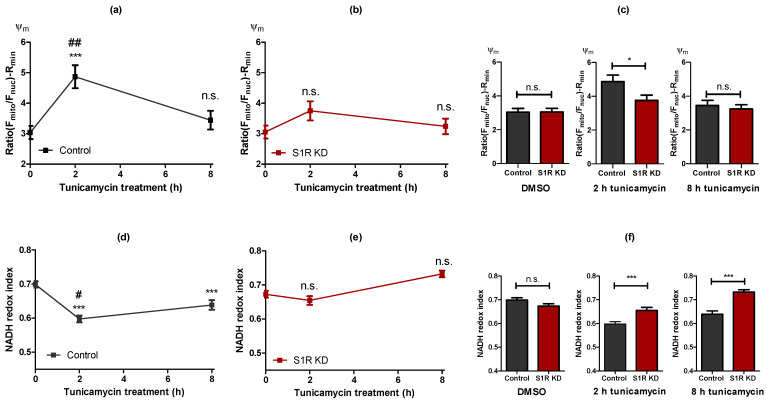
Alterations in the mitochondrial bioenergetic status are largely dependent on S1R during early ER stress. Time course of Ψ_m_ after tunicamycin treatment, presented as mean ± SEM and assessed by the mitochondrial to nucleus TMRM fluorescence ratio in control SH-SY5Y cells (**a**) and in cells with S1R KD (**b**). Bar graphs represent MEAN ± SEM of Ψ_m_ in control (black) and S1R KD (red) cells before tunicamycin treatment (left), after 2 h (middle), or 8 h (right) tunicamycin treatment (**c**). Time course of NADH redox index after tunicamycin treatment, presented as MEAN ± SEM and assessed NADH autofluorescence in control SH-SY5Y cells (**d**) and in cells with S1R KD (**e**). Bar graphs represent MEAN ± SEM of NADH redox index in control (black) and S1R KD (red) cells before tunicamycin treatment (left), after 2 h (middle), or 8 h (right) tunicamycin treatment (**f**). One-way ANOVA with Tukey’s multiple comparison test (**a**,**b**,**d**,**e**), *** p < 0.001, ## *p* < 0.01 and # *p* < 0.05 (control 2 h tunicamycin against control 8hr tunicamycin), n.s.—not significant; unpaired t-test (**c**,**f**), *** p < 0.001, * p < 0.05, n.s.—not significant; Ψ_m_: Control DMSO (57 cells/10 experiments), Control 2hr tunicamycin (31 cell/5 experiments), Control 8hr tunicamycin (42 cells/7 experiments), S1R KD DMSO (54 cells/10 experiments), S1R KD 2 h tunicamycin (29 cells/5 experiments), S1R KD 8 h tunicamycin (40 cells/7 experiments); NADH redox index: Control DMSO (208 cells/9 experiments), Control 2 h tunicamycin (203 cell/6 experiments), Control 8 h tunicamycin (143 cells/6 experiments), S1R KD DMSO (243 cells/9 experiments), S1R KD 2hr tunicamycin (111 cells/5 experiments), S1R KD 8hr tunicamycin (129 cells/6 experiments).

Next, we investigated mitochondrial membrane potential (Ψ_m_) using tetramethylrhodamine methyl ester (TMRM), a cationic fluorescent dye that accumulates in mitochondria in a membrane potential-dependent manner (Figure S2a). Ψ_m_ serves as a marker for mitochondrial bioenergetic status and was increased after 2 h of tunicamycin treatment, and restored to control levels after 8 h of treatment (Figure 3a). The increase in Ψ_m_ after 2 h was significantly lower in S1R KD cells in comparison to control cells (Figure 3b,c). The effect of S1R antagonist BD1047 was comparable to that of the siRNA-mediated KD (Figure S2b,c). Similar to mitochondrial ATP data, Ψ_m_ emphasizes the importance of S1R for mitochondrial bioenergetics during the initial steps of ER stress.

We next evaluated mitochondrial NADH redox index [16] as an additional readout for mitochondrial bioenergetics, measured as mitochondrial NADH autofluorescence normalized to the minimum and maximum values achieved by uncoupling and blocking of NADH dehydrogenase complex, respectively. NADH redox index was decreased by 2 h of tunicamycin treatment and less so by 8 h of treatment (Figure 3d), which indicates more energized mitochondria. Since the NADH redox index represents NADH/NAD^+^ ratio, the decline of this ratio, combined with increased mitochondrial membrane potential and ATP, means more flux of electron donors (NADH) to the electron transport chain (ETC). S1R KD abolished this reduction of the NADH redox index at both time points (Figure 3e). The difference between the control group and the S1R knock-down was only apparent during ER stress induction by tunicamycin treatment (Figure 3f).

Since tunicamycin-induced alterations in mitochondrial ATP, Ψ_m_, and NADH redox index were abolished by S1R KD and/or the usage of an S1R antagonist, we assume S1R to serve as a key player in controlling mitochondrial bioenergetics during ER stress. Hence, because the 2 h treatment with tunicamycin resulted in a pronounced boost in mitochondrial bioenergetics, and this increase is approaching baseline status after 8 h of ER stress, S1R seems to be crucial, particularly during the early phase of ER stress. 

### 2.4. Early ER Stress Gives Rise to an Increased ER Ca^2+^ Flux That Is Directed towards Mitochondria by Sigma-1 Receptor

To clarify the mechanism of how S1R is facilitating the increase of mitochondrial bioenergetics during early ER stress, we investigated mitochondria-associated ER membranes (MAMs). Although increased MAMs were reported for HeLa cells after 4 h of ER stress [5], we observed no change in the MAMs of tunicamycin treated control cells or the respective S1R knock-down cells (Figure S3a,b). 

**Figure 4:**
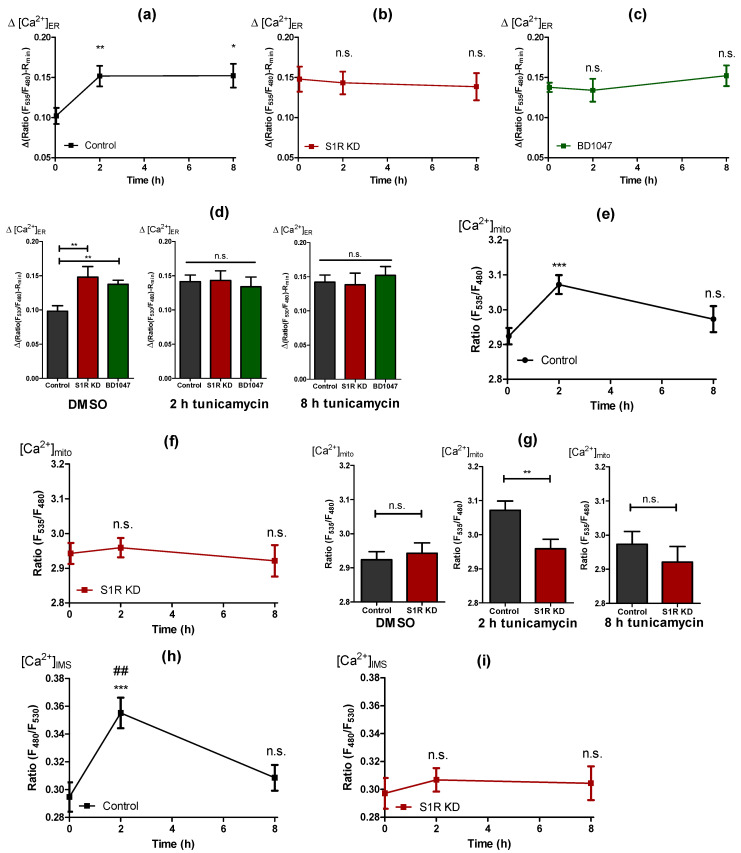

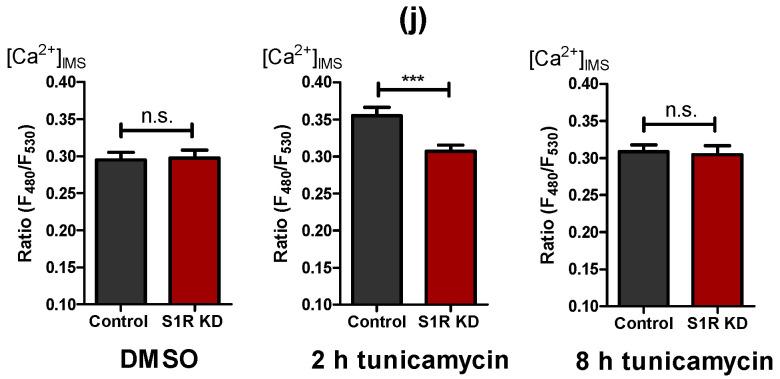
Cells develop an enhanced ER Ca^2+^ leak that is directed towards mitochondria by S1R during early ER stress. Time course of ER Ca^2+^ leak after tunicamycin treatment, presented as mean ± SEM and assessed by change in normalized D1ER ratio in control (**a**), S1R KD (**b**) and BD1047 treated (**c**) SH-SY5Y cells. Bar graphs represent MEAN ± SEM of ER Ca^2+^ leak in control (black), S1R KD (red) and BD1047 treated (green) cells before tunicamycin treatment (left), after 2 h (middle), or 8 h (right) tunicamycin treatment (**d**). Time course of mitochondrial Ca^2+^ level after tunicamycin treatment, presented as MEAN ± SEM and assessed by 4mtD3cpv ratio in control SH-SY5Y cells (**e**) and in cells with S1R KD (**f**). Bar graphs represent MEAN ± SEM of mitochondrial Ca^2+^ levels in control (black) and S1R KD (red) cells before tunicamycin treatment (left), after 2 h (middle), or 8 h (right) tunicamycin treatment (**g**). Time course of IMS Ca^2+^ level after tunicamycin treatment, presented as MEAN ± SEM and assessed by IMS-GEM-GECO ratio in control SH-SY5Y cells (**h**) and in cells with S1R KD (**i**). Bar graphs represent MEAN ± SEM of IMS Ca^2+^ levels in control (black) and S1R KD (red) cells before tunicamycin treatment (left), after 2 h (middle), or 8 h (right) tunicamycin treatment (**j**). One-way ANOVA with Tukey’s multiple comparison test (**a**–**c**,**e**,**f**,**h**,**i**), *** p < 0.001, ** p < 0.01, * p < 0.05, ## *p* < 0.01 (for h, control 2 h tunicamycin against control 8 h tunicamycin), n.s.—not significant; unpaired t-test (**d**,**g**,**j**), *** p < 0.001, ** p < 0.01, * p < 0.05, n.s.—not significant; ER Ca^2+^ leak: Control DMSO (43 cells/15 experiments), Control 2 h tunicamycin (44 cell/16 experiments), Control 8 h tunicamycin (38 cells/12 experiments), S1R KD DMSO (19 cells/9 experiments), S1R KD 2 h tunicamycin (27 cells/10 experiments), S1R KD 8 h tunicamycin (19 cells/6 experiments), BD1047 DMSO (23 cells/11 experiments), BD1047 2 h tunicamycin (9 cells/6 experiments), BD1047 8 h tunicamycin (18 cells/6 experiments); Mitochondrial Ca^2+^: Control DMSO (38 cells/14 experiments), Control 2 h tunicamycin (40 cell/13 experiments), Control 8 h tunicamycin (29 cells/8 experiments), S1R KD DMSO (35 cells/14 experiments), S1R KD 2 h tunicamycin (38 cells/12 experiments), S1R KD 8 h tunicamycin (25 cells/8 experiments); IMS Ca^2+^: Control DMSO (37 cells/4 experiments), Control 2 h tunicamycin (35 cell/4 experiments), Control 8 h tunicamycin (28 cells/3 experiments), S1R KD DMSO (38 cells/4 experiments), S1R KD 2 h tunicamycin (39 cells/4 experiments), S1R KD 8 h tunicamycin (36 cells/4 experiments).

Further on, we investigated whether ER stress is associated with an ER Ca^2+^ leak by using genetically-encoded ER Ca^2+^ probe D1ER [17]. Thereby, the ER Ca^2+^ leak was estimated by the change in ER Ca^2+^ level in response to the removal of extracellular Ca^2+^, which excludes the possibility of ER Ca^2+^ refilling. ER Ca^2+^ leak was increased by tunicamycin treatment for 2 and 8 h in control cells (Figure 4a). Interestingly, ER Ca^2+^ leak remained unaffected by tunicamycin treatment in S1R KD (Figure 4b), and BD1047 treated (Figure 4c) cells, but was found to be elevated in untreated S1R KD and BD1047 cells compared to controls (Figure 4d, left panel). In addition to the ER Ca^2+^ leak, we observed an unexpected increase in basal ER Ca^2+^ load with 2 and 8 h of tunicamycin treatment (Figure S4a). Similar to the leak, basal ER Ca^2+^ of S1R KD cells did not change with tunicamycin treatment (Figure S4b) and was higher in non-treated S1R KD cells compared to controls (Figure S4d, left panel), whereas the basal ER Ca^2+^ of BD1047 treated cells did not differ from controls (Figure S4c,d).

The increase in ER Ca^2+^ leak in response to tunicamycin could explain increased mitochondrial bioenergetics in control cells, since the increased leak would supply mitochondria with more Ca^2+^ under these conditions. However, since a similarly strong ER Ca^2+^ leak was also established by S1R knock-down and BD1047 treatment, but without changes in mitochondrial bioenergetics, further experiments were necessary to clarify these differences. 

Thus, we also quantified basal mitochondrial Ca^2+^ level using the genetically encoded mitochondria-targeted Ca^2+^ biosensor, 4mtD3cpv [18]. We observed increased mitochondrial Ca^2+^ levels in control cells in response to tunicamycin treatment (Figure 4e). Interestingly, mitochondrial Ca^2+^ levels were not significantly affected by S1R knock-down cells with or without tunicamycin treatment (Figure 4f,g) despite the increased ER Ca^2+^ leak (Figure 4b,d). These results point to a possible function of S1R during early ER stress, which is directing the enhanced ER Ca^2+^ leak towards sites of mitochondrial Ca^2+^ uptake, while the knock-down of S1R yields an undirected ER Ca^2+^ leak, which is less sensed by mitochondria. The fact that ER Ca^2+^ leak was not changed between 2 h and 8 h of tunicamycin treatment (Figure 4a), but mitochondrial Ca^2+^ level is diminished from 2 h to 8 h of ER stress (Figure 4e), points to a possibility that S1R’s “directing” activity of ER Ca^2+^ leak is a time-dependent phenomenon. The diminishing of the mitochondrial Ca^2+^ increase after 8 h of ER stress also explains the return of mitochondria to the basal bioenergetic state observed in control cells (Figure 2a and Figure 3a,d). 

To test whether the observed differences in mitochondrial Ca^2+^ were due to the direction of the ER Ca^2+^ leak and not associated with Ca^2+^ “permeability” of the mitochondrial inner membrane, we measured Ca^2+^ levels in the mitochondrial inter-membrane space (IMS) using a recently developed IMS-targeted genetically encoded ratiometric Ca^2+^ sensor, IMS-GEM-GECO [19,20]. IMS Ca^2+^ closely followed the same trends as the mitochondrial Ca^2+^, with an increase after 2 h of tunicamycin treatment, which was normalized after 8 h of treatment (Figure 4h and Figure S5a). In S1R depleted and BD1047 treated cells, tunicamycin treatment did not elevate IMS Ca^2+^ after 2 h and 8 h (Figure 4i,j and Figure S5b,c), thus supporting our assumption of a specific role of S1R as an orchestrator of the enhanced ER Ca^2+^ leak during early ER stress, directing the Ca^2+^ leak towards mitochondrial Ca^2+^ uptake sites.

### 2.5. Sigma-1 Receptor Protects against Increased Mitochondrial ROS Production during ER Stress

**Figure 5:**
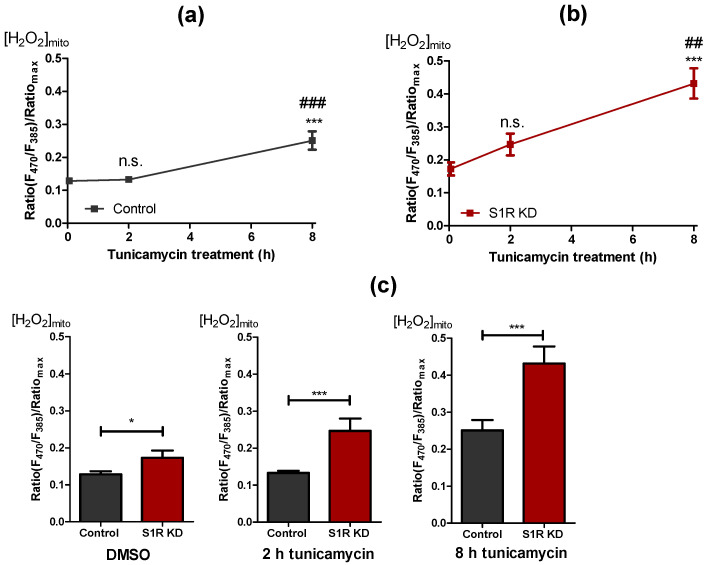
S1R KD is accompanied by increased ROS levels. Time course of mitochondrial H_2_O_2_ levels after tunicamycin treatment, presented as mean ± SEM and assessed by the mitoHyPer7 in control SH-SY5Y cells (**a**) and in cells with S1R KD (**b**). Bar graphs represent mean ± SEM of mitochondrial H_2_O_2_ levels in control (black) and S1R KD (red) cells before tunicamycin treatment (left), after 2 h (middle), or 8 h (right) tunicamycin treatment (**c**). Comparison of the mitochondrial ROS level in control and S1R KD cells at corresponding treatment durations (error bars are SEM); One-way ANOVA with Tukey’s multiple comparison test (**a**,**b**), *** p < 0.001, *## p* < 0.01 (for b, S1R KD 8 h against S1R KD 2 h), ### p < 0.001 (for a, control 8 h against control 2 h), n.s.—not significant; unpaired t-test (**c**), * p < 0.05, *** p < 0.001; Control DMSO (22 cells/7 experiments), Control 2 h tunicamycin (32 cell/10 experiments), Control 8 h tunicamycin (22 cells/6 experiments), S1R KD DMSO (19 cells/9 experiments), S1R KD 2 h tunicamycin (27 cells/10 experiments), S1R KD 8 h tunicamycin (19 cells/6 experiments).

Along with Ca^2+^ signaling and energy metabolism, mitochondrial reactive oxygen species (ROS) production plays a significant part in mitochondrial and cellular fitness under ER stress [21,22]. As it has been reported that S1R has an important role in mitochondrial ROS metabolism [8,23], we investigated the contribution of S1R to mitochondrial ROS production in our model of early ER stress in SH-SY5Y neuroblastoma cells. We monitored mitochondrial ROS using the genetically encoded mitochondrial ROS sensor, mitoHyper7 [24]. Induction of ER stress in control cells with tunicamycin for 2 h did not yield a change in mitochondrial ROS, whereas after 8 h of treatment, mitochondrial ROS levels significantly increased (Figure 5a). On the other hand, tunicamycin treatment resulted in a reasonably linear increase in mitochondrial ROS in S1R knock-down cells, with drastically increased ROS levels after 8 h of treatment (Figure 5b). Basal mitochondrial ROS, as well as ROS levels after 2 h and 8 h of tunicamycin treatment, were increased by S1R knock-down (Figure 5c). These results indicate that S1R prevents mitochondrial ROS production during early ER stress.

## 3. Discussion

Since ER stress and UPR are hallmarks of many human pathologies, including neurodegeneration and cancer [9,11], we have attempted to clarify the involvement of S1R in mitochondrial bioenergetics during the early phases of ER stress. We have induced ER stress in SH-SY5Y neuroblastoma cells with tunicamycin treatment for 2 and 8 h and validated it by quantifying XBP1 splicing (Figure 1a,b). Our data did not support previously reported findings that S1R affects XBP1 splicing [8], which is likely explained by the low knock-down efficiency of S1R in our model system (Figure S1a). Low knock-down efficiency is stemming out of a low transfection rate (10%) and the absence of a marker for transfected cells in the qPCR analysis used to quantify XBP1 splicing. When the knock-down efficiency was assessed in co-transfected GFP-positive sorted cells, S1R mRNA level was reduced by 75% (Figure S1b). Consequently, the absence of a significant difference in XBP1 splicing between control and S1R knock-down cells is not definitive. A slight reduction of XBP1 splicing in S1R KD cells was observed after 8 h of tunicamycin treatment and might indicate the reduced IRE1 activity because of S1R knock-down. For all the remaining experiments, we have inclined towards single-cell measurements. 

Having established ER stress induction over time in vitro, we could demonstrate that mitochondrial bioenergetics are getting substantially augmented after 2 h of ER stress (Figure 2a and Figure 3a,d). S1R seems to play a major role in this adaptation, since knock-down and a pharmacological antagonist of S1R almost eliminated respective responses (Figure 2b,c and Figure 3b,e and Figure S2c). In search for a possible mechanism of action of S1R, we measured the amount of MAMs, as it has been reported that MAMs are increasing during early or later stages of ER stress [5,25]. Our results in SH-SY5Y cells showed no detectable changes in MAMs after 2 h and 8 h of tunicamycin treatment in control or S1R knock-down cells (Figure S3a,b). The possible reasons for discrepancies with published data could be specificities of cell lines used, as most of the reported studies were performed in HeLa cells [5,25]. 

Next, we looked directly into ER Ca^2+^ leak, since it can influence mitochondrial energetics during ER stress and was previously reported to be increased during ER stress [26,27]. In line with these reports, we have observed increased ER Ca^2+^ leak after 2 h and 8 h of tunicamycin treatment in control cells (Figure 4a). Still, surprisingly, a similar leak was present in untreated S1R knock-down and BD1047 treated cells, which did not change with tunicamycin treatment (Figure 4b–d). This was a puzzling finding, since the increased leak in S1R KD and BD1047 treatment did not fit together with mitochondrial energetics data, unless the Ca^2+^ leak was not directed towards mitochondria in knock-down BD1047 treated cells.

We could elaborate supportive data for the latter as mitochondrial, and IMS Ca^2+^ levels were increased after 2 h of tunicamycin treatment in control and not in S1R KD and antagonist treated cells (Figure 4e–j and Figure S5a–c), despite the presence of comparable ER Ca^2+^ leak after 2 h and 8 h of ER stress in all conditions. The prevalence of the increased Ca^2+^ level in the IMS during early ER stress in control and not in S1R KD and BD1047 treated cells validates the driving role of S1R directed ER Ca^2+^ leak as a promoter of mitochondrial bioenergetics and removes the possibility of hampered permeability of mitochondrial inner membrane as a result of reduced level or activity of S1R. Additionally, since the reduction of S1R expression by siRNA or the activity by the antagonist on its own was enough to trigger ER Ca^2+^ leak (Figure 4d, left panel) that was not directed to mitochondria, it is easy to speculate that S1R is essential for ER homeostasis under unstressed conditions as well.

In support of our findings, a recent study elegantly showed a UPR-independent function of IRE1, where it serves as a scaffold for IP_3_R at the MAMs [28], and another study showed that S1R stabilizes IRE1 during ER stress at the MAM region [8]. Together with these interesting reports, our current work points towards the function of S1R as a director of ER Ca^2+^ towards mitochondria during early ER stress as a mechanism to increase mitochondrial bioenergetics to eventually supply more ATP for UPR. Diminishing mitochondrial ATP and bioenergetics after 8 h of ER stress (Figure 2a and Figure 3a,d) despite the same enhanced ER Ca^2+^ leak (4a) in control cells adds an extra argument in support of time dependency of S1R orchestrated ER Ca^2+^ leak directed at mitochondria. In support of this claim, it was previously shown that S1R redistributes from the MAM region towards the remaining parts of the ER after prolonged ER stress [7], hence its ER leak directing function would be decreasing as ER stress progresses further. As a result, the ER Ca^2+^ leak would no longer be pointed towards mitochondria, as evidenced by a drop of the mitochondrial and IMS Ca^2+^ levels back to basal levels after 8 h of ER stress (Figure 4e,h).

Although the matter of ER Ca^2+^ leak directed towards sites of mitochondrial Ca^2+^ uptake by S1R to enhance mitochondrial bioenergetics to supply ATP for ER UPR seems fitting to the overall picture, the molecular identity of protein(s) responsible for the S1R controlled mitochondria-directed ER Ca^2+^ leak is not clear. We are tempted to speculate that IP_3_R3 is the likely candidate, but this claim needs further investigation. Our findings of increased basal ER Ca^2+^ levels that we observed in S1R knock-down cells and control cells treated with tunicamycin are not clear and need further attention. One possible explanation might be that S1R antagonizes store-operated Ca^2+^ entry (SOCE) [29]; hence the knock-down of S1R might result in increased ER Ca^2+^ level as a result of increased SOCE. Additionally, increased mitochondrial ATP production during early ER stress (Figure 2a) could enhance ER Ca^2+^ sequestration through sarco(endo)plasmic reticulum Ca^2+^-ATPase (SERCA) activity.

By increasing mitochondrial bioenergetics during early ER stress, S1R maintains a supply of ATP to fuel UPR to restore ER homeostasis, but in the meanwhile, it is known that enhanced oxidative phosphorylation can generate ROS. To compensate for this, S1R shows the ability to moderate excessive ROS production, as we have demonstrated S1R’s importance in maintaining a balanced ROS metabolism under resting and ER stress conditions (Figure 5). These findings are supported by published data emphasizing the involvement of S1R in protection against oxidative stress and provide a possible mechanism for enhanced cell death of S1R deficient cells undergoing ER stress [7,8,23]. Taken together, by simultaneously enhancing mitochondrial bioenergetics and reducing ROS generation, S1R acts as a pro-survival agent for a cell facing ER stress. 

In conclusion, we have demonstrated that S1R plays a crucial role during the early stages of ER stress, whereby it promotes mitochondrial bioenergetics by directed Ca^2+^ mobilization and protects against mitochondrial ROS elevation. We provide novel mechanistic insights into the complex ER-to-mitochondria communication during the onset of ER stress which might have multiple implications in different human pathologies, and highlight the need for further studies involving pre-clinical disease models involving ER stress, such as Alzheimer’s disease and other neurodegenerative disorders. 

## 4. Materials and Methods

List of abbreviations and sensors used in the study.
**Abbreviation****Expanded Version**
EREndoplasmic reticulum
UPRUnfolded protein response
S1RSigma-1 Receptor
PERKDouble-stranded RNA-dependent protein kinase (PRK)-like ER kinase
ATF6Activating transcription factor 6
IRE1Inositol requiring enzyme 1
ERADEndoplasmic-reticulum-associated protein degradation
MAMsMitochondria-associated ER membranes
IP_3_R3Inositol 1,4,5-trisphosphate receptor
XBP1X-box binding protein 1
KDKnock-down
siRNASmall interfering RNA
GFPGreen fluorescent protein
DMSODimethyl sulfoxide
ATPAdenosine triphosphate
FRETFörster resonance energy transfer
Ψ_m_Mitochondrial membrane potential
NADHNicotinamide adenine dinucleotide
IMSMitochondrial inter-membrane space
ROSReactive oxygen species
SERCASarco(endo)plasmic reticulum Ca^2+^-ATPase
**Sensor****Definition****Reference**mtAT1.03Genetically encoded mitochondrial matrix targeted FRET-based ATP biosensor[14]TMRMTetramethylrhodamine methyl ester
D1ERGenetically encoded ER targeted FRET-based Ca^2+^ biosensor[17]4mtD3cpvGenetically encoded mitochondrial matrix targeted FRET-based Ca^2+^ biosensor[18]IMS-GEM-GECOGenetically encoded mitochondrial IMS targeted ratiometric Ca^2+^ biosensor[19,20]mitoHyper7Genetically encoded mitochondrial matrix targeted ratiometric ROS biosensor[24]

### 4.1. Cell Culture and Transfection

Human neuroblastoma SH-SY5Y cells (Sigma-Aldrich, catalogue number 94030304, lot number 18I031, passage number 11) were grown in Dulbecco’s Modified Eagle’s Medium (DMEM) (Sigma-Aldrich; Vienna, Austria) containing 10% FCS, penicillin (100 U/mL), streptomycin (100 µg/mL), amphotericin (1.25 µg/mL), 1 g/L glucose and 4 mM glutamine in a humidified incubator (37 °C, 5% CO_2_, 95% air). Cells were used until passage 20. Cells were tested for mycoplasma contamination and were negative. For all microscopy experiments with S1R knock-down (siRNA sequence: 5′-GCU CAC CAC CUA CCU CUU UdTdT-3′) with or without genetically encoded sensors, cells were plated on 30 mm glass coverslips and transfected at 60–80% confluence with siRNA using 3 µL of TransFast transfection reagent (Promega, Madison, WI, USA) in 1 mL serum- and antibiotic-free medium with or without 1 µg plasmid DNA encoding an appropriate sensor for 10–16 h. Afterward, the transfection media was replaced by a full culture medium. All experiments were performed 40–48 h after transfection. Prior to experiments, cells were adjusted to room temperature and shortly kept in experimental storage buffer (2 mM Ca^2+^, 138 mM NaCl, 1 mM MgCl_2,_ 5 mM KCl, 10 mM HEPES, 2.6 mM NaHCO_3_, 0.44 mM KH_2_PO_4_, amino acid, and vitamin mix, 10 mM glucose, 2 mM L-glutamine, 1% Penicillin/Streptomycin, 1% Fungizone, pH adjusted to 7.4).

Tunicamycin (0.6 µM, 1/10.000 dilution from DMSO stock) or DMSO (1/10.000 dilution) were added to the culture medium for indicated times, and treated cells were incubated in the humidified incubator. BD1047 (Cat. No. 0956, Tocris, Abingdon, UK) was dissolved in water and used at a final concentration of 10 µM and added along with tunicamycin or DMSO, and was also present in the experimental storage buffer.

### 4.2. Quantitative PCR and XBP1 Splicing

Total mRNA was isolated using RNeasy^®^ Mini Kit (Qiagen, Hilden, Germany), and reverse transcription was done using Applied Biosystems High-Capacity cDNA Reverse Transcription kit (Thermo Fisher Scientific Baltics UAB, Vilnus, Lithuania). qPCR was performed using Promega GOTaq^®^ qPCR Master Mix (Madison, WI, USA). Knock-down efficiency was determined using specific primers for S1R (Forward: CACTCGGGGCGCTACTG; reverse: TGTACTACCGTCTCCCCTGG) and normalized to HPRT1 and GAPDH (for sorted cells). Spliced XBP1 was analyzed with specific primers (forward: GCTGAGTCCGCAGCAGGT; reverse: CTGGGTCCAAGTTGTCCAGAAT). Spliced XBP1s were normalized to HPRT1.

### 4.3. Live Cell Imaging

All live-cell microscopy experiments were performed on an Olympus IX73 inverted microscope if not mentioned otherwise. The microscope is equipped with an UApoN340 40× oil immersion objective (Olympus, Tokyo, Japan) and a CCD Retiga R1 camera (Q-imaging, Tucson, AZ, Canada). For illumination, a LedHUB^®^ (Omicron, Vienna, Germany) equipped with 340, 385, 455, 470, and 550 nm LEDs in combination with CFP/YFP/RFP (CFP/YFP/mCherry-3X, Semrock, Rochester, NY, USA) or GFP (GFP-3035D, Semrock, Rochester, NY, USA) filter set was used. Alternatively, an AnglerFish F-G/O (NGFI, Grraz, Austria) has been used. Data acquisition and control of the fluorescence microscope were performed using Visiview 4.2.01 (Visitron, Puchheim, Germany). 

### 4.4. Mitochondrial ATP, Membrane Potential and NADH Redox Index Measurements

Mitochondrial ATP was measured using genetically encoded mitochondrial matrix targeted ATP sensor AT1.03 [14] (gift from Hiromi Imamura, Kyoto University, Kyodai Graduate School of Biostudies, Japan). The sensor was excited with 455 nm LED and emission collected at 480 nm and 530 nm using a CFP/YFP/RFP filter set and 505dcxr beam-splitter. Background-subtracted emission ratio of 530/480 was analyzed.

Mitochondrial membrane potential was measured using tetramethylrhodamine methyl ester (TMRM). TMRM was excited with 550 nm LED and emission collected at 600 nm using CFP/YFP/RFP filter set. Cells were incubated with 20 nM TMRM in an experimental storage buffer for 20 min at room temperature. During the measurement, cells were perfused with physiological buffer (2 mM Ca^2+^, 135 mM NaCl, 1 mM MgCl_2_, 5 mM KCl, 10 mM HEPES, 10 mM glucose, pH adjusted to 7.4) using a gravity-based perfusion system (NGFI, Graz, Austria). After the baseline recording, cells were perfused with physiological buffer containing 1 µm FCCP to fully depolarize mitochondria to obtain the minimum values. Background-subtracted TMRM fluorescence ratio of mitochondrial to nucleus region was used as readout.

Mitochondrial NADH autofluorescence was monitored using 340 nm LED as previously described [16]. Shortly, SH-SY5Y cells were perfused with physiological buffer to record baseline reading, followed by perfusion with 0.5 µM FCCP until the signal reached the minimum and flatted, and then perfused with 2 µM rotenone until a plateau is reached. Mitochondrial NADH redox index was quantified as background-subtracted baseline autofluorescence normalized by subtracting the minimum value reached by FCCP and divided by the maximum value reached by rotenone.

### 4.5. ER Ca^2+^ Measurements

ER Ca^2+^ was measured with genetically encoded ER Ca^2+^ sensor D1ER [17] on an inverted wide field microscope (Observer.A1, Carl Zeiss GmbH, Vienna, Austria) equipped with a 40x objective (Plan Apochromat 1,3 NA Oil DIC (UV) VIS-IR, Carl Zeiss GmbH, Vienna, Austria) and a standard CFP/YFP filter cube. D1ER was excited with 425 nm and emission collected with 505dcxr beam-splitter on two sides of the camera (CCD camera, Coolsnap Dyno, Photometrics, Tucson, AZ, USA). Data acquisition and control of the fluorescence microscope setup were performed using the NIS-Elements AR software (Nikon, Vienna, Austria). Basal D1ER emission ratio (emission 530 nm/480 nm) was recorded for 2 min, while the cells were perfused with physiological buffer followed by 8 min perfusion with physiological buffer without Ca^2+^ (138 mM NaCl, 1 mM MgCl_2_, 5 mM KCl, 10 mM HEPES, 0.1 mM EGTA, 10 mM glucose, pH adjusted to 7.4). After this, the ER Ca^2+^ was emptied by perfusing the cells with 4 µM ionomycin in Ca^2+^ free buffer. Background-subtracted emission ratio of D1ER was normalized to the minimum ratio reached by ionomycin. ER Ca^2+^ leak was quantified as the D1ER ratio drop after 8 min of perfusion with Ca^2+^ free buffer.

### 4.6. ER-Mitochondria Co-Localization

ER was labeled with D1ER and mitochondria stained with TMRM (50 nM). Cells were imaged on a confocal spinning disk microscope (Axio Observer.Z1 from Zeiss, Gottingen, Germany) equipped with 100× objective lens (Plan-Fluor x100/1.45 Oil, Zeiss), a motorized filter wheel (CSUX1FW, Yokogawa Electric Corporation, Tokyo, Japan) on the emission side, AOTF-based laser merge module for laser line 405, 445, 473, 488, 561, and 561 nm (Visitron Systems) and a Nipkow-based confocal scanning unit (CSU-X1, Yokogawa Electric corporation). The D1ER and TMRM were alternately excited with 488 and 561 nm laser lines, respectively, and emissions were acquired at 530 and 600 nm using a charged CCD camera (CoolSNAP-HQ, Photometrics, Tucson, AZ, USA). Z-stacks of both channels in 0.2 µm increments were recorded. VisiView acquisition software (Universal Imaging, Visitron Systems) was used to acquire the imaging data. Images were blindly deconvoluted with NIS-elements v5.1 (Nikon, Vienna, Austria). The colocalization was determined on a single-cell level using ImageJ and the plugin coloc2. The Pearson coefficient was calculated.

### 4.7. Mitochondrial and IMS Ca^2+^ Measurements

Mitochondrial Ca^2+^ was measured using genetically encoded matrix targeted Ca^2+^ sensor 4mtD3cpv [18]. The sensor was excited with 455 nm LED and emission collected at 480 nm and 530 nm using a CFP/YFP/RFP filter set and 505dcxr beam-splitter. Background-subtracted emission ratio of 530 nm/480 nm was analyzed.

IMS Ca^2+^ was measured using genetically encoded IMS targeted Ca^2+^ sensor IMS-GEM-GECO [19,20]. The sensor was excited with 385 nm LED and emission collected at 480 nm and 530 nm using a CFP/YFP/RFP filter set and 505dcxr beam-splitter. Background-subtracted emission ratio of 480 nm/530 nm was analyzed.

### 4.8. Mitochondrial ROS Measurements

Mitochondrial ROS was measured using genetically encoded matrix targeted ROS sensor mitoHyper7 [24]. The sensor was excited with 385 nm and 470 nm LEDs and emission collected with GFP filter set. After recording the baseline, the cells were perfused with 200 µM H_2_O_2_ to get the maximum readout. The background-subtracted and normalized ratio of 470 nm/385 nm was analyzed.

### 4.9. Data Analysis

The number of independent experiments is indicated in each figure legend along with the used statistical test and *p* value. For single-cell experiments, cells were used for analysis. Statistical analyses, including Student’s t-test and Analysis of variance (ANOVA) with Tukey post hoc test, were performed on GraphPad Prism software version 5.04 (GraphPad Software, San Diego, CA, USA) and Microsoft Excel (Microsoft Office 2013).

## Data Availability

The data presented in this study are available on request from the corresponding author and also contained within the main article and supplements.

## Supplementary Materials

The following are available online at https://www.mdpi.com/article/10.3390/metabo11070422/s1, Figure S1: Knock-down efficiency of S1R siRNA in unsorted (a) and sorted (b) cells determined by qRT-PCR and normalized to housekeeping gene, Figure S2: Protocol for Ψm measurements and the impact of BD1047 on Ψm, Figure S3: Tunicamycin treatment and S1R KD do not affect ER-mitochondria co-localization in SH-SY5Y neuroblastoma cells, Figure S4: Tunicamycin treatment and S1R KD increase the basal ER Ca2+ level, Figure S5: Time course of IMS Ca2+ level after tunicamycin treatment.
