# Supplementary material for: Sigma-1 Receptor Promotes Mitochondrial Bioenergetics by Orchestrating ER Ca2+ Leak during Early ER Stress
Source: Metabolites. 2021 Jun 26;11(7):422. doi: 10.3390/metabo11070422 (PMC8305890; doi:10.3390/metabo11070422)
Supplement: Supplementary file 1 [file metabolites-11-00422-s001.zip › metabolites-1243987-supplementary.pdf]

Supplementary Materials:

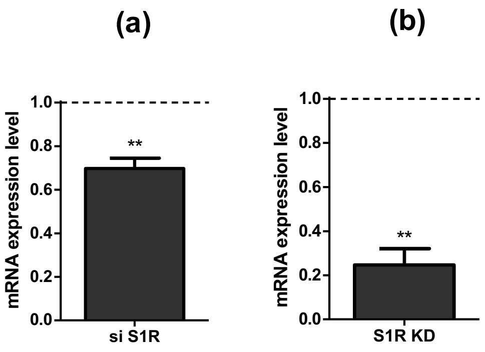

Figure S1. Knock-down efficiency of S1R siRNA in unsorted **(a)** and sorted **(b)** cells determined by qRT-PCR and normalized to housekeeping gene. Paired t-test, **p<0.01, n=5 **(a)**, n=3 **(b)**.

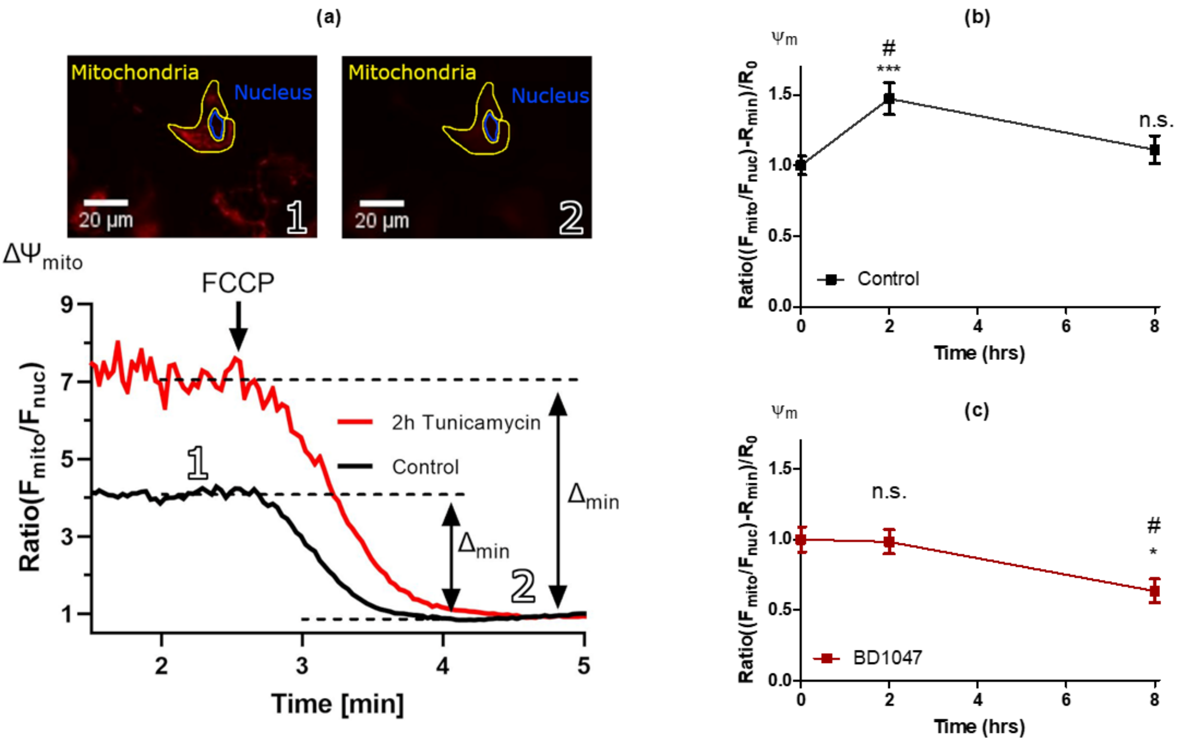

Figure S2. Protocol for Ψ_m measurements and the impact of BD1047 on Ψ_m. (**a**) Examplary images of SH-SY5Y cells stained with TMRM (red) and selected regions of interest (ROI) for mitochondria and nucleus. Experimental protocol used to asses Ψ_m is shown below for 2 cells, one treted with DMSO (black line) and the other with tunicamycin for 2 hours (red line). Change in the TMRM fluorescence ratio of mitochondrial to nucleus ROI from basal to after FCCP treatment was used to estimate Ψ_m. Time course of Ψ_m after tunicamycin treatment, normalized to respective DMSO treated controls and presented as mean ± SEM in control **(b)** and BD1047 treated **(c)** cells. One-way ANOVA with Tukey's multiple comparison test, ***p<0.001, *p<0.05, #p<0.05 (2 h tunicamycin against 8hr tunicamycin), n.s. – not significant; Control DMSO (62 cells/10 experiments), Control 2hr tunicamycin (37 cell/5 experiments), Control 8hr tunicamycin (42 cells/7 experiments), BD1047 DMSO (33 cells/5 experiments), S1R KD 2 h tunicamycin (26 cells/4 experiments), S1R KD 8 h tunicamycin (23 cells/4 experiments);

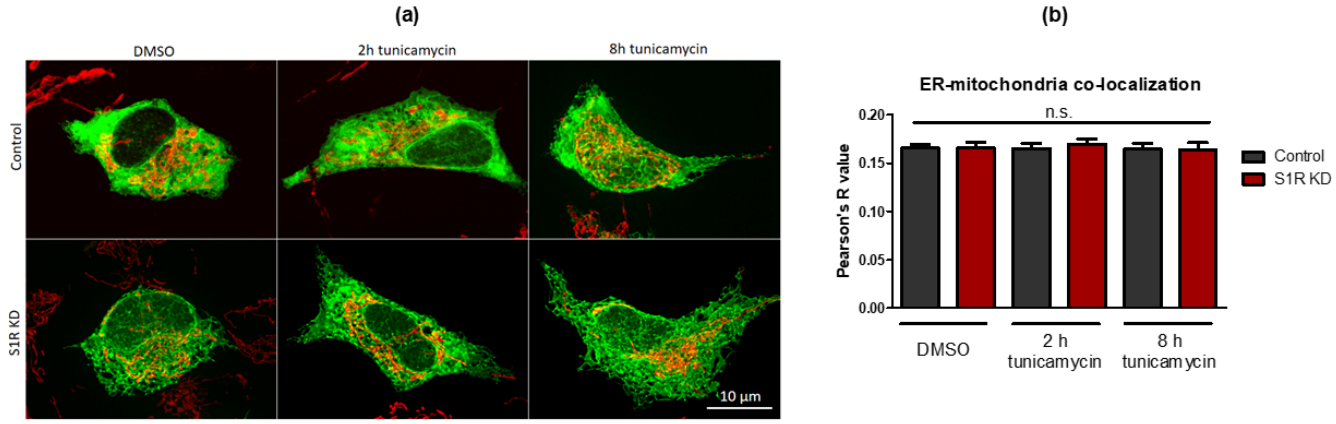

Figure S3. Tunicamycin treatment and S1R KD do not affect ER-mitochondria co-localization in SH-SY5Y neuroblastoma cells. **(a)** Representative z-projections of 3D-stacks of SH-SY5Y cells with 200 nm increments are shown; ER is labelled with D1ER (green) and mitochondria with TMRM (red). (**b**) Bar graphs represent MEAN ± SEM of ER-mitochondrial co-localization represented by Pearson's R value. One-way ANOVA with Tukey's multiple comparison test, n.s. – not significant; Control DMSO (158 cells/16 experiments), Control 2hr tunicamycin (90 cell/9 experiments), Control 8hr tunicamycin (76 cells/8 experiments), S1R KD DMSO (119 cells/12 experiments), S1R KD 2hr tunicamycin (90 cells/9 experiments), S1R KD 8hr tunicamycin (74 cells/8 experiments).

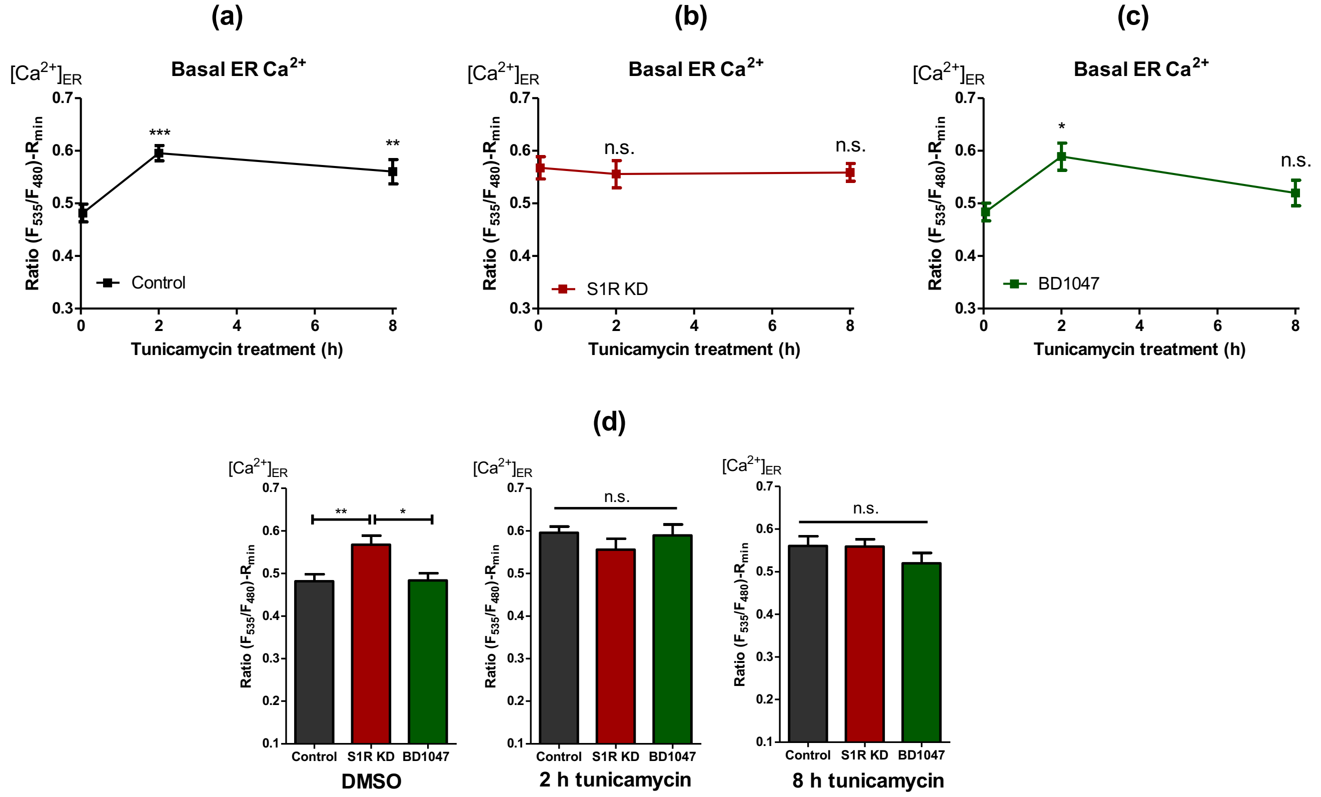

Figure S4. Tunicamycin treatment and S1R KD increase the basal ER Ca^{2+} level. Time course of ER Ca^{2+} level after tunicamycin treatment, presented as mean ± SEM and assessed by normalized D1ER ratio in

control SH-SY5Y cells **(a)**, in cells with S1R KD **(b)** and BD1047 treatment **(c)**. Bar graphs represent MEAN ± SEM of ER Ca^{2+} in control (black), S1R KD (red) and BD1047 treated (green) cells before tunicamycin treatment (left), after 2 h (middle) or 8 h (right) tunicamycin treatment **(d)**. One-way ANOVA with Tukey's multiple comparison test, ***p<0.001, **p<0.01, *p<0.05, n.s. – not significant; Control DMSO (43 cells/15 experiments), Control 2hr tunicamycin (44 cell/16 experiments), Control 8hr tunicamycin (38 cells/12 experiments), S1R KD DMSO (19 cells/9 experiments), S1R KD 2hr tunicamycin (27 cells/10 experiments), S1R KD 8hr tunicamycin (19 cells/6 experiments), BD1047 DMSO (23 cells/11 experiments), BD1047 2hr tunicamycin (9 cells/6 experiments), BD1047 8hr tunicamycin (18 cells/6 experiments).

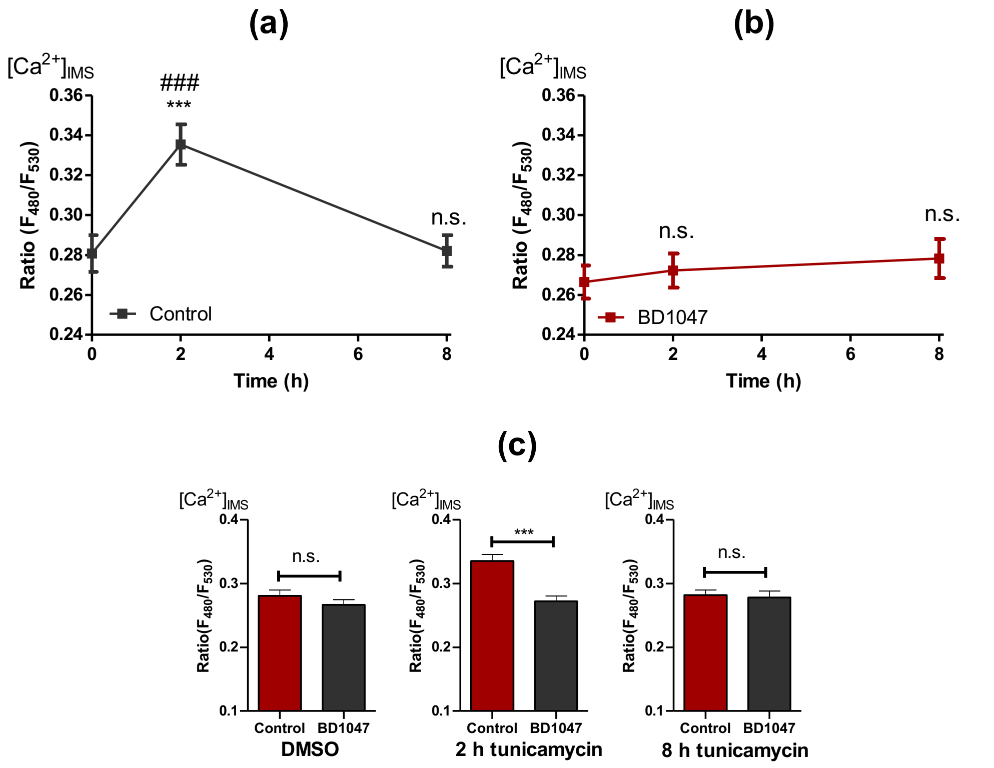

Figure S5. Time course of IMS Ca^{2+} level after tunicamycin treatment, presented as MEAN ± SEM and assessed by IMS-GEM-GECO ratio in control SH-SY5Y cells **(a)** and in cells treated with BD1047 **(b)**. Bar graphs represent MEAN ± SEM of IMS Ca^{2+} levels in control (black) and BD1047 treated (red) cells before tunicamycin treatment (left), after 2 h (middle) or 8 h (right) tunicamycin treatment **(c)**. One-way ANOVA with Tukey's multiple comparison test **(a-b)**, ***p<0.001, ###p<0.001 (control 2 h tunicamycin against control 8 h tunicamycin), n.s. – not significant; unpaired t-test **(c)**, ***p<0.001, n.s. – not significant; Control DMSO (86 cells/9 experiments), Control 2 h tunicamycin (72 cell/8 experiments), Control 8 h tunicamycin (71 cells/8 experiments), BD1047 DMSO (61 cells/7 experiments), BD1047 2 h tunicamycin (71 cells/7 experiments), BD1047 8hr tunicamycin (63 cells/7 experiments).
